# Factors associated with depression in older adults during the COVID-19 pandemic: findings from The Irish Longitudinal Study on Ageing

**DOI:** 10.3389/fpsyt.2026.1799213

**Published:** 2026-04-22

**Authors:** Xiang Jiao, Wei Wang, Lina Zhou

**Affiliations:** Department of Psychiatry, The First Affiliated Hospital of Xi’an Jiaotong University, Xi’an, Shaanxi, China

**Keywords:** COVID-19, depression, intervention strategies, lifestyles, TILDA

## Abstract

**Objectives:**

The coronavirus disease 2019 (COVID-19) pandemic has adversely affected the physical and mental health of older adults worldwide. This study aimed to identify the factors associated with depression among older adults during the pandemic and to develop strategies to enhance their psychological well-being.

**Methods:**

Data from The Irish Longitudinal Study on Ageing (TILDA) collected during the COVID-19 pandemic were compared with pre-pandemic data (Wave 5). We examined the impact of the pandemic on depressive symptoms in older adults and investigated potential influencing factors related to depression, including lifestyle changes, COVID-19 concern and protective behaviors, and psychological assessments.

**Results:**

The prevalence of clinically significant depressive symptoms in older adults was significantly higher after the onset of the COVID-19 pandemic than in the pre-pandemic Wave 5 (p < 0.001). The rates of difficulty falling asleep and early awakening increased significantly, whereas the time spent on anaerobic exercise, aerobic exercise, and slow walking decreased markedly (all p < 0.001). Hierarchical multiple linear regression analysis revealed that the model including predictors such as age, gender, Perceived Stress Scale score, UCLA Loneliness Scale score, difficulty falling asleep, early awakening, sleep duration, days of anaerobic exercise, days of aerobic exercise, and days of slow walking was statistically significant (F = 165.241, p < 0.001, R = 0.734, R² = 0.539, adjusted R² = 0.536), explaining 53.9% of the variance in depressive symptoms.

**Conclusion:**

The COVID-19 pandemic might be associated with an increase in depressive symptoms among older adults. This exacerbation is closely linked to lifestyle changes (sleep disturbances and reduced physical activity), psychological factors (heightened stress and loneliness), and certain information-seeking behaviors. These findings underscore the need for integrated interventions that target these modifiable risk factors.

## 1 Introduction

Since its emergence in December 2019, the coronavirus disease 2019 (COVID-19) pandemic has impacted global health. Beyond its direct threat to physical well-being, the pandemic has also precipitated a crisis in mental health (1). These adverse psychological effects stem not only from the pathological consequences of the infection and its associated stress but also from widespread public health measures, including isolation, reduced social contact, and decreased physical activity (2, 3). Such reductions in social interaction and activity are particularly detrimental to older adults, as they are strongly linked to depression and other negative emotions in this population (4, 5). Consequently, the pandemic may have exacerbated pre-existing vulnerabilities, thereby triggering or worsening poor mental health outcomes in older adults (2, 6).

Numerous studies have documented elevated rates of depression, anxiety, stress, and insomnia during this period. For instance, Shah et al. (7) found that more than half of the study participants reported symptoms of anxiety (50.9%), stress (57.4%), and depression (58.6%). Another study reported severe depression in 17.0% and severe anxiety in 14.8% of its 666 respondents (8). Depression, a prevalent mental illness among older adults, is of particular concern. Our previous research has demonstrated that depressive symptoms can significantly impair cognitive function and the ability to perform daily activities in older adults (9, 10). Therefore, understanding the specific effects of the pandemic on depressive symptoms in this vulnerable group is crucial for safeguarding their health and well-being.

A growing body of evidence suggests that pandemic-related lifestyle changes are closely associated with psychological distress. Shah et al. (7) noted a correlation between lack of exercise and increased stress, anxiety, and depression. A narrative review on social isolation during COVID-19 highlighted anxiety, depression, poor sleep quality, and physical inactivity as key adverse outcomes for older adults (3). Similarly, Gorenko et al. (11) argued that physical distancing measures, while necessary to curb viral transmission, increased the risk of social isolation and loneliness—factors known to be associated with anxiety, depression, cognitive decline, and even mortality. Other factors reported to correlate with depressive symptoms during the pandemic include altered patterns of physical activity and sleep, increased consumption of convenience foods, changes in alcohol and tobacco use, and reduced use of public transportation (12, 13). Psychological factors such as loneliness (14) and perceived stress (12), along with a perceived decline in quality of life (15), have also been implicated. Conversely, access to accurate, up-to-date health information and adherence to precautionary measures like hand hygiene and mask-wearing have been linked to lower levels of psychological distress (16), suggesting that health-related awareness and protective behaviors may serve as protective factors.

In summary, the effects of COVID-19 on depressive symptoms in older adults are likely multifaceted, involving a complex interplay of lifestyle changes, physical activity, health-related awareness and behaviors, and psychological factors. While previous studies have identified various correlates of depression during the pandemic, few have utilized large-scale community data to examine the specific changes in depressive symptoms among older adults from the pre- to post-pandemic period. Given the profound and lasting effects of the pandemic on this population’s lives, the present study aims to fill this gap. We analyzed data from a large community-based survey to (1) compare the prevalence and severity of depressive symptoms in older adults before and after the pandemic; and (2) identify key social, behavioral, and psychological factors associated with these changes. The findings will provide a robust evidence base for developing targeted, effective mental health support strategies to improve the overall well-being of the aging population.

## 2 Methods

### 2.1 Study design and participants

The Irish Longitudinal Study on Ageing (TILDA) collects comprehensive information on the health, economic, and social circumstances of adults aged 50 years and older residing in Ireland, using data collected in biennial waves. For the present study, we included data from two time points, Wave 5 (collected from January 2018 to January 2019) and the COVID-19 Sub-Study (collected from July to November 2020), representing the periods before and after the onset of the COVID-19 pandemic.

TILDA Wave 5 includes 4,960 respondents and covers domains such as aging, cognition, economic status, health, lifestyle, mental health, retirement, and social participation. The COVID-19 Sub-Study focuses on adults aged at least 60 years during the early months of the pandemic. Participants completed the COVID-19 Science Creative Quarterly (SCQ), which captures experiences related to COVID-19, medical care, health, lifestyle, information sources, social participation, preventive behaviors, family support, income, employment, and related topics. A total of 3,773 participants returned the SCQ, yielding a response rate of 68% over five months.

Inclusion criteria for the COVID-19 Sub-Study were: (1) age ≥ 60 years; (2) complete demographic data; (3) completion of COVID-19-related questionnaires, including items on concern about COVID-19, protective measures, lifestyle changes, daily exercise, and sleep during the pandemic; (4) complete data for the Center for Epidemiological Studies Depression Scale (CESD-8), Perceived Stress Scale (PSS), and UCLA Loneliness Scale; and (5) no reported COVID-19 infection. For Wave 5, inclusion criteria were: (1) age ≥ 60 years; (2) complete demographic data; (3) complete data on daily exercise and sleep; and (4) complete data for the CESD-8, PSS, and UCLA Loneliness Scale. To ensure that the ages of patients in the two cohorts were equivalent, we restricted the age to 60 years and above when defining the inclusion criteria for Wave 5 because the COVID-19 Sub-Study group included only individuals of this age bracket.

**Figure 1:**
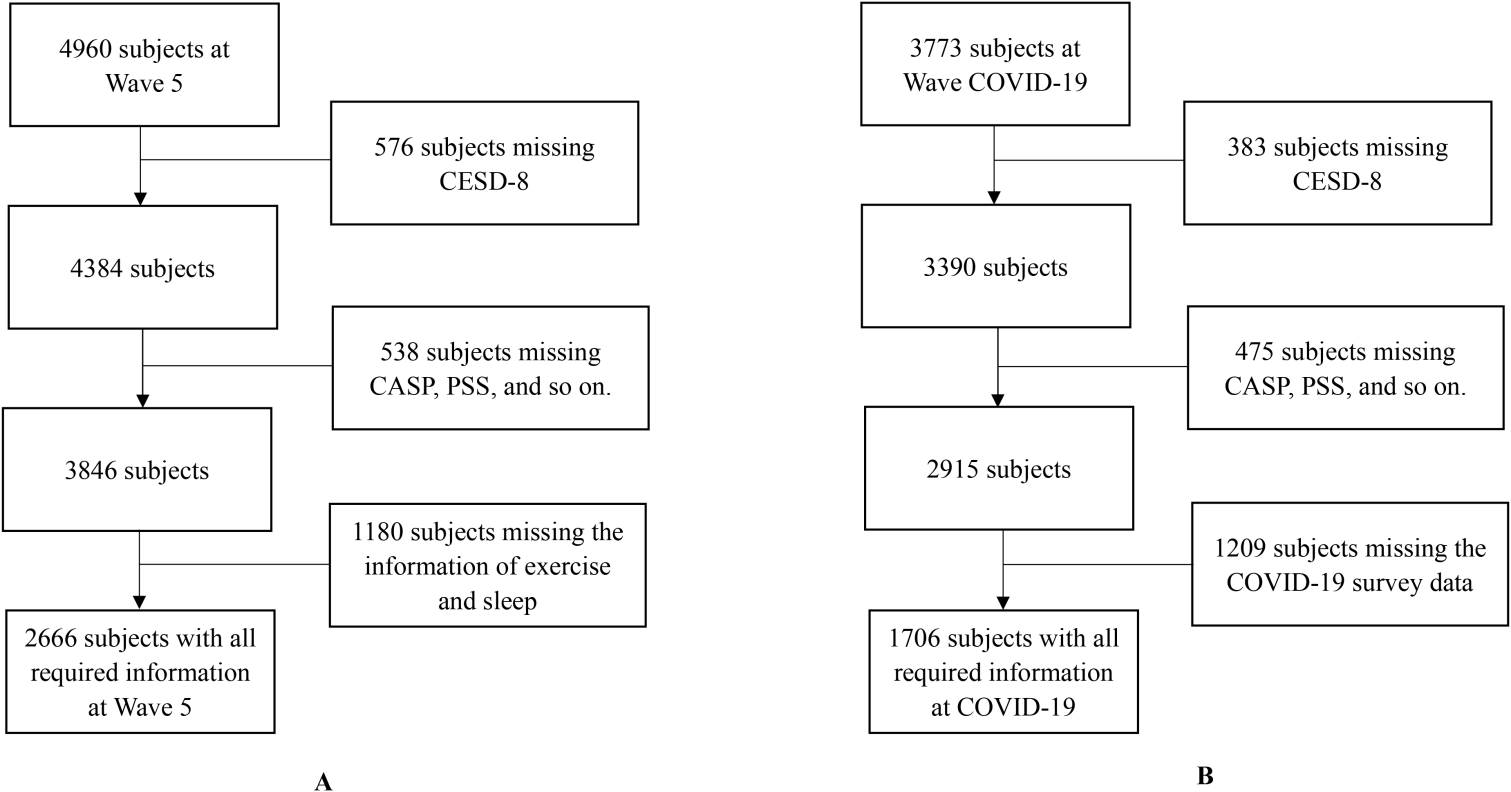
Flow chart of the selection of the study population for Wave 5 **(A)** and COVID-19 **(B)**.

The participant selection process is summarized in **Figure 1**. The final analytical sample consisted of 2,666 participants from Wave 5 and 1,706 from the COVID-19 Sub-Study, but the participants in these two waves are not identical.

### 2.2 Lifestyle assessment

Lifestyle was assessed using a questionnaire that asked respondents: “Since the outbreak of the COVID-19 pandemic, how often have you done the following activities compared to before the outbreak?” Each item was rated on a 4-point scale: not at all, less often, about the same, and more often. The activities assessed included smoking; drinking; leaving home; grocery shopping; visiting family; visiting friends; attending religious services; exercising at home; walking outside for > 20 min; engaging in hobbies, crafts, or puzzles; watching TV or streaming content; volunteering; gardening or home repairs; reading print or online materials; participating in social groups via video conferences; and financial situation.

### 2.3 COVID-19 concern and protective behaviors

A study-specific questionnaire assessed the following: (1) frequency of COVID-19 news consumption (several times a day, once per day, less than once per day, or never), (2) sources of news (national radio, Facebook, local radio, Irish television, Twitter, WhatsApp, other television, national/local newspapers in print, or online), and (3) level of concern about the pandemic on a scale from 1 (least concerned) to 10 (most concerned). The number of news channels used was also calculated.

Protective behaviors were assessed with five yes-or-no items asking whether respondents had washed their hands more frequently, used hand sanitizers or disinfectants, paid special attention to covering coughs or sneezes, taken medication to prevent COVID-19, or worn a face mask outside the home.

### 2.4 Measures of loneliness

The UCLA Loneliness Scale was used to assess loneliness among older adults. The original UCLA scale has 20 items and assesses loneliness resulting from the gap between an individual’s desire for social interaction and their actual level (17). Five items were selected for the large-scale census. Previous studies have confirmed that even using only four α coefficients can obtain better α coefficients. Each item contains three options, which are divided into hardly ever or never, some of the time, and often according to their frequency, with scores ranging from 0 to 2, respectively. The higher the total score, the stronger the loneliness.

### 2.5 Measures of perceived stress

The perceived stress of the study participants was assessed using the 4-item PSS (18). The PSS uses a 5-point scale, with each item ranging from 0 to 4, indicating the range from never to very much. The higher the scale score, the higher the perceived stress.

### 2.6 Measures of depressive symptoms

Depressive symptoms were assessed using the 8-item CESD-8, derived from the 20-item CESD scale. CESD-8 evaluates the performance in the past week. According to the duration of the performance, each item was divided into less than 1 day, 1–2 days, 3–4 days, and 5–7 days, with a score from 0 to 3, respectively. Higher scores indicate more severe depressive symptoms. Previous studies have verified that the CESD-8 has good reliability and validity in large-scale population surveys of older adults (19). Based on previous TILDA research reports, a score ≥ 9 was used in the present study to define cases with clinically significant depressive symptoms. This threshold has demonstrated good sensitivity and specificity in TILDA (20).

### 2.7 Data analysis

All analyses were conducted using SPSS 26.0. Continuous variables are presented as means ± standard deviations, and categorical variables as counts and percentages. The present analysis adopts a repeated cross-sectional design. Group comparisons (Wave 5 vs. COVID-19 Sub-Study) were performed using independent sample *t*-tests or non-parametric alternatives for continuous variables and *chi*-square tests for categorical variables.

To examine the differences in mental health and exercise between the two waves, we compared CESD-8, UCLA Loneliness Scale, and PSS scores. We then compared lifestyle factors, COVID-19-related concerns, and protective behaviors between the depressed and non-depressed groups and selected variables with significant differences for further analysis. Finally, to identify factors associated with depressive symptoms during COVID-19, we conducted a theoretically guided hierarchical multiple linear regression with the CESD-8 score as the dependent variable, entering significant predictors from prior comparisons. Predictors were entered in four blocks based on their conceptual proximity to depressive symptoms: 1. Block 1 (Demographics): age, gender, and lives alone. 2. Block 2 (Core Psychosocial Factors): PSS score and UCLA Loneliness Scale score. 3. Block 3 (Health Behaviors): difficulty falling asleep, early awakening, anaerobic exercise, aerobic exercise, slow walking, and sleep time. 4. Block 4 (Pandemic-Specific Factors): change in drinking; smoking; listening to the national radio; exercising at home; walking outside for > 20 min; engaging in hobbies, crafts, or puzzles; watching TV, Netflix, streaming movies, or shows; doing garden work or home repairs; reading books, magazines, or newspapers; frequency of handwashing; level of concern about the COVID-19; reading related news from Facebook, local radio, or national newspapers; and financial situation. Variance inflation factors were examined to check for multicollinearity. To assess the stability and generalizability of the final model, we performed bootstrap validation (1,000 samples) to derive robust confidence intervals for the regression coefficients and conducted a 70/30 split-sample cross-validation.

## 3 Results

### 3.1 Changes in mental health and physical activity among older adults after the onset of the COVID-19 pandemic

Data from Wave 5 and the COVID-19 Sub−Study were analyzed to compare the mental health and lifestyle profiles of two independent cohorts of older adults: one assessed before the pandemic (Wave 5) and one assessed during the pandemic (COVID-19 Sub−Study). After adjusting for age and gender differences between the two respondent groups, the prevalence of clinically significant depressive symptoms was significantly higher in the COVID−19 Sub-study group than in the Wave 5 group (16.88% vs. 6.49%, p < 0.001). Scores on the CESD−8, UCLA Loneliness Scale, and PSS were also significantly elevated in the COVID−19 Sub-Study group (all p < 0.05). Additionally, sleep quality and physical activity deteriorated after the onset of the pandemic. Specifically, the incidence rates of difficulty falling asleep and early awakening were significantly increased in the COVID−19 Sub-Study group compared to the Wave 5 group, whereas the time spent in anaerobic exercise, aerobic exercise, and slow walking decreased markedly (all p < 0.001). These results are presented in **Table 1A**.

**Table 1A:** Changes in mental health and physical activity among older adults after the onset of the COVID-19 pandemic.

Variables		Wave5 (n=2666)	COVID-19 (n=1706)	t/c^2^	P
Gender	Men, N, %	1234(46.29)	765(44.84)	0.875	**0.035**
	Women, N, %	1432(53.71)	941(55.16)		
Age, year, M ± SD		68.10 ± 7.645	68.78 ± 7.074	-2.935	**0.003**
CESD, M ± SD		2.89 ± 3.321	4.67 ± 3.995	-15.937	**<0.001**
UCLA-LS, M ± SD		1.50 ± 1.976	3.17 ± 1.882	-27.756	**<0.001**
PSS, M ± SD		3.70 ± 2.944	4.28 ± 2.769	-6.49	**<0.001**
Anaerobic exercise, Day, M ± SD		1.72 ± 0.981	1.39 ± 2.084	4.089	**<0.001**
Aerobic exercise, Day, M ± SD		3.20 ± 2.016	2.22 ± 2.494	11.556	**<0.001**
Slow walking, Day, M ± SD		7.15 ± 5.369	5.06 ± 2.327	15.025	**<0.001**
Difficulty falling asleep	Yes, N, %	1138(42.69)	890(52.17)	37.618	**<0.001**
	No, N, %	1528(57.31)	816(47.83)		
Early awaking	Yes, N, %	743(27.87)	1092(64.01)	557.875	**<0.001**
	No, N, %	1923(72.13)	614(35.99)		

M ± SD, Mean ± standard deviation; CESD, the Center for Epidemiological Studies Depression Scale; UCLA-LS, the UCLA Loneliness Scale; PSS, Perceived Stress Scale.

Bold values indicate statistically significant results (p < 0.05).

Based on CESD−8 scores (cutoff ≥ 9), COVID−19 respondents were classified into a depression group (n = 288) and a control group (n = 1,418). Compared with the control group, the depression group had a higher proportion of women; higher PSS and UCLA Loneliness Scale scores; less time spent in aerobic, anaerobic, and slow walking activities; shorter sleep duration; and higher rates of difficulty falling asleep and early awakening. These findings are summarized in **Table 1B**.

**Table 1B:** Differences in mental health and exercise among older adults with and without depression during the COVID-19 epidemic.

Variables			Control group (n=1418)	Depression group (n=288)	t/c^2^	df	P
Age, year, M ± SD			68.78 ± 7.008	68.75 ± 7.404	0.064	1704	0.949
Gender		Men, N, %	670(47.25)	95(32.99)	19.69	1	**<0.001**
		Women, N, %	748(52.75)	193(60.01)			
Lives alone		Yes, N, %	283(19.96)	81(28.13)	9.514	1	**0.002**
		No, N, %	1135(80.04)	207(71.87)			
Education		Primary education, N, %	142(10.01)	29(10.07)	0.07	2	0.965
		Secondary education, N, %	573(40.41)	114(39.58)			
		Higher Education, N, %	703(49.58)	145(50.35)			
CESD, M ± SD			3.25 ± 2.437	11.65 ± 2.573	-52.838	1704	**<0.001**
UCLA-LS, M ± SD			2.79 ± 1.574	5.04 ± 2.139	-16.961	1704	**<0.001**
PSS, M ± SD			3.60 ± 2.406	7.20 ± 2.582	-21.3	1704	**<0.001**
Anaerobic exercise, Day, M ± SD			1.47 ± 2.122	0.96 ± 1.835	4.187	1704	**<0.001**
Aerobic exercise, Day, M ± SD			2.33 ± 2.521	1.70 ± 2.288	4.173	1704	**<0.001**
Slow walking, Day, M ± SD			5.19 ± 2.234	4.45 ± 2.659	4.403	1704	**<0.001**
Sleep time, Hour, M ± SD			7.19 ± 1.56	6.61 ± 1.657	5.736	1704	**<0.001**
Difficulty falling asleep	Rarely/never		759(53.53)	57(19.79)	186.486	2	**<0.001**
	Sometimes		586(41.33)	156(54.17)			
	Most of the time		73(5.15)	75(26.04)			
Early awaking	Rarely/never		568(40.06)	46(15.97)	181.032	2	**<0.001**
	Sometimes		734(51.76)	139(48.26)			
	Most of the time		116(8.18)	103(35.76)			

M ± SD, Mean ± standard deviation; CESD, the Center for Epidemiological Studies Depression Scale; UCLA-LS, the UCLA Loneliness Scale; PSS, Perceived Stress Scale.

Bold values indicate statistically significant results (p < 0.05).

### 3.2 Lifestyle factors and depression

To avoid distortions due to responses of “*never*”, such responses were excluded from percentage calculations in both groups; detailed data are shown in **Table 2**. The results indicated that, compared with the control group, the depression group showed significantly greater reductions in smoking and more frequent increases or decreases in alcohol consumption after the pandemic (all p < 0.05).

**Table 2:** Lifestyle changes of depressed individuals after the onset of the COVID-19 Pandemic.

Variables		N(%)		t/c2	df	P
		Control group (n=1418)	Depression group (n=288)			
Smoking	Total	89	37	5.51	2	**0.019**
	Less	22(24.72)	17(45.95)			
	Same	67(75.28)	20(54.05)			
	More	0(0.00)	0(0.00)			
Drinking	Total	1092	213	13.662	2	**0.001**
	Less	294(26.92)	73(34.27)			
	Same	621(56.87)	92(43.19)			
	More	177(16.21)	48(22.54)			
Leave your home	Total	1309	256	1.966	2	0.374
	Less often	1089(83.19)	222(86.72)			
	About the same	208(15.89)	32(12.50)			
	More often	12(0.92)	2(0.78)			
Go grocery shopping	Total	1105	215	0.48	2	0.787
	Less often	875(79.19)	166(77.21)			
	About the same	209(18.91)	44(20.47)			
	More often	21(1.90)	5(2.33)			
Travel to visit family members	Total	625	118	0.742	2	0.690
	Less often	574(91.84)	111(94.07)			
	About the same	45(7.20)	7(5.93)			
	More often	6(0.96)	2(1.69)			
Travel to visit friends	Total	350	73	4.868	2	0.088
	Less often	333(95.14)	69(94.52)			
	About the same	17(4.86)	3(4.11)			
	More often	0(0.00)	1(1.37)			
Attend religious services outside your home	Total	165	41	0.481	2	0.488
	Less often	120(72.73)	32(78.05)			
	About the same	45(27.27)	9(21.95)			
	More often	0(0.00)	0(0.00)			
Exercise at home	Total	1183	214	7.982	2	**0.018**
	Less often	170(14.37)	47(21.96)			
	About the same	704(59.51)	117(54.67)			
	More often	309(26.12)	50(23.36)			
Walk outside your home for more than 20 minutes	Total	1312	245	15.976	2	**<0.001**
	Less often	293(22.33)	81(33.06)			
	About the same	544(41.46)	100(40.82)			
	More often	475(36.20)	64(26.12)			
Do hobbies, crafts, or puzzles	Total	1189	215	24.599	2	**<0.001**
	Less often	120(10.09)	47(21.86)			
	About the same	641(53.91)	106(49.30)			
	More often	428(36.00)	62(28.84)			
Watch TV, Netflix, stream movies, or shows	Total	1378	274	26.883	2	**<0.001**
	Less often	89(6.46)	16(5.84)			
	About the same	807(58.56)	117(42.70)			
	More often	482(34.98)	141(51.46)			
Volunteer	Total	354	51	3.569	2	0.168
	Less often	148(41.81)	16(31.37)			
	About the same	180(50.85)	28(54.90)			
	More often	26(7.34)	7(13.73)			
Do garden work or home repairs	Total	1317	249	12.202	2	**0.002**
	Less often	78(5.92)	29(11.65)			
	About the same	481(36.52)	95(38.15)			
	More often	758(57.56)	125(50.20)			
Read books, magazines, or newspapers	Total	1373	262	10.93	2	**0.004**
	Less often	49(3.57)	18(6.87)			
	About the same	769(56.01)	123(46.95)			
	More often	555(40.42)	121(46.18)			
Meet with social groups on Zoom or other online video conference sites	Total	719	100	0.997	2	0.607
	Less often	74(10.29)	13(13.00)			
	About the same	174(24.20)	26(26.00)			
	More often	471(65.51	61(61.00)			
Financial situation	Much worse off	29(2.05)	14(4.86)	10.084	4	**0.018**
	A little worse off	133(9.38)	29(10.07)			
	About the same	899(63.40)	188(65.28)			
	A little better off	322(22.71)	52(18.06)			
	Much better off	35(2.47)	5(1.74)			

Bold values indicate statistically significant results (p < 0.05).

Regarding sleep, the depression group had a significantly higher incidence of difficulty falling asleep and early awakening, as well as a shorter sleep duration than the control group (all p < 0.001).

In terms of physical activity, a significantly larger proportion of the depression group reported exercising at home and walking outside for > 20 min less often than before the pandemic, whereas a significantly smaller proportion reported exercising at home or walking outside more often than before (all p < 0.05). Furthermore, the depression group had fewer days per week of anaerobic exercise, aerobic exercise, and walking than the control group (all p < 0.001).

Concerning leisure activities, participants with depressive symptoms were less likely than controls to report spending more time on hobbies, watching movies or TV, gardening or home repairs, and reading, and they were more likely to report spending less time on these activities (all p < 0.05).

Financially, fewer individuals in the depression group reported an improved financial situation, and more reported a worsened financial situation, relative to the control group (all p < 0.05).

### 3.3 Association of depression with COVID-19 concerns and protective behaviors

No difference was found between the depression and control groups in the frequency of following COVID−19 news (p > 0.05). However, the sources of news differed: a larger proportion of depressed individuals obtained COVID−19 news via local radio and Facebook, whereas a smaller proportion used national newspapers (all p < 0.05). An interesting, although non-significant, trend was observed for national radio (p = 0.052), with fewer depressed individuals using this source. The self-rated attention to COVID−19 news was significantly higher in the depression group than in the control group (all p < 0.05). The results are summarized in **Table 3**.

**Table 3:** Differences in COVID-19 concerns and protection measures.

Variables			Control group (n=1418)	Depression group (n=288)	t/c2	df	P
			N(%)	N(%)			
Frequency of reading related news	Several times a day		781(55.08)	156(54.17)	3.149	3	0.369
	Once per day		586(41.33)	111(38.54)			
	Less than once per day		59(4.16)	18(6.25)			
	Never		10(0.71)	3(1.04)			
National radio		Yes	1012(71.37)	189(65.63)	3.789	1	0.052
		No	406(28.63)	99(34.38)			
Facebook		Yes	164(11.57)	51(17.71)	8.201	1	**0.004**
		No	1254(88.43)	237(82.29)			
Local radio		Yes	324(22.85)	83(28.82)	4.697	1	**0.030**
		No	1094(77.15)	205(71.18)			
Irish television		Yes	1093(77.08)	209(72.57)	2.695	1	0.101
		No	325(22.92)	79(27.43)			
Twitter		Yes	38(2.68)	9(3.13)	0.177	1	0.674
		No	1380(97.32)	279(96.88)			
WhatsApp		Yes	117(8.25)	27(9.38)	0.391	1	0.532
		No	1301(91.75)	261(90.63)			
Other television		Yes	605(42.67)	111(38.54)	1.672	1	0.196
		No	813(57.33)	177(61.46)			
www.gov.ie		Yes	144(10.16)	34(11.81)	0.698	1	0.404
		No	1274(89.84)	254(88.19)			
www.hse.ie		Yes	168(11.85)	38(13.19)	0.409	1	0.522
		No	1250(88.15)	250(86.81)			
National newspapers		Yes	728(51.34)	119(41.32)	9.615	1	**0.002**
		No	690(48.66)	169(58.68)			
Local newspapers		Yes	237(16.71)	56(19.44)	1.255	1	0.263
		No	1181(83.29)	232(80.56)			
The level of concern about the COVID-19			7.93 ± 1.918	8.39 ± 1.874	-3.692	1704	**<0.001**
Frequency of hand washing		Yes	1405(99.08)	276(95.83)	19.008	1	**<0.001**
		No	12(0.85)	12(4.17)			
Using disinfectant		Yes	1300(91.68)	272(94.44)	2.53	1	0.112
		No	118(8.32)	16(5.56)			
Protect against coughing and sneezing		Yes	1394(98.31)	282(97.92)	0.212	1	0.645
		No	24(1.69)	6(2.08)			
Taking medication to prevent COVID-19		Yes	64(4.51)	17(5.90)	1.022	1	0.312
		No	1354(95.49)	271(94.10)			
Wearing a mask when going out		Yes	1063(74.96)	211(73.26)	0.366	1	0.545
		No	355(25.04)	77(26.74)			

Bold values indicate statistically significant results (p < 0.05).

Among those who reported increased handwashing after the pandemic, the proportion of individuals with depressive symptoms was lower than that in the control group. No significant differences were observed in other protective measures, including the use of disinfectants, covering coughs or sneezes, taking preventive medications, and wearing masks outside the home (all p > 0.05).

### 3.4 Factors associated with depression after the onset of the COVID-19 pandemic

In the full analytical sample (n=1,706), a multivariable linear regression analysis with theoretically guided hierarchical selection was performed using the CESD−8 score as the dependent variable. The optimal model included the following predictors: age, gender, PSS score, UCLA Loneliness Scale score, difficulty falling asleep, early awakening, sleep time, anaerobic exercise days, aerobic exercise days, and slow walking days. The model was statistically significant (F = 165.241, p < 0.001, R = 0.734, R² = 0.539, adjusted R² = 0.536), explaining 53.9% of the variance in depressive symptoms. The results are summarized in **Table 4**. Bootstrap validation with 1,000 replications indicated that the 95% confidence intervals for the regression coefficients were closely aligned with those obtained from the original model, supporting the stability and robustness of our estimates. Cross-validation results indicated stable model performance. The model trained on the training subset (n = 1,157) yielded an R² of 0.551. When applied to the held-out validation subset (n = 549), it explained an equivalent proportion of variance (predicted R² = 0.525). The minimal decrease in predictive power suggests that the model generalizes well and shows no substantial evidence of overfitting.

**Table 4:** Factors associated with depression during the COVID-19 epidemic.

Variables	B	95%CI	β	t	P	Tolerance	VIF
Age	0.008	-0.011,0.027	0.014	0.831	0.406	0.955	1.047
Gender	0.01	-0.264,0.284	0.001	0.072	0.943	0.898	1.114
PSS	0.563	0.51,0.616	0.39	20.879	**<0.001**	0.779	1.283
UCLA Loneliness Scale	0.673	0.598,0.749	0.317	17.528	**<0.001**	0.831	1.203
Difficulty falling asleep (sometimes)	0.915	0.612,1.217	0.114	5.932	**<0.001**	0.742	1.347
Difficulty falling asleep (most of the time)	2.056	1.478,2.633	0.145	6.982	**<0.001**	0.632	1.583
Early awaking (sometimes)	0.562	0.254,0.869	0.07	3.581	**<0.001**	0.706	1.417
Early awaking (most of the time)	2.08	1.562,2.597	0.174	7.883	**<0.001**	0.557	1.795
Sleep time	-0.009	-0.131.0.113	-0.003	-0.148	0.882	0.693	1.444
Anaerobic exercise, Day	-0.041	-0.111,0.029	-0.021	-1.154	0.249	0.79	1.266
Aerobic exercise, Day	-0.002	-0.06,0.056	-0.001	-0.08	0.936	0.797	1.255
Slow walking, Day	-0.105	-0.163.-0.031	-0.061	-3.614	**<0.001**	0.943	1.06

Bold values indicate statistically significant results (p < 0.05).

## 4 Discussion

This study compared data from TILDA Wave 5 with those from the TILDA COVID−19 Sub−Study, revealing a significant increase in clinically significant depressive symptoms among older adults during the pandemic, accompanied by heightened loneliness, greater perceived stress, and reduced sleep and physical activity. To identify factors associated with this increase in depression, we compared lifestyle characteristics between depressed and non−depressed older adults in the COVID−19 sample. Individuals with depression were more likely to report a reduction in smoking, an increase or decrease in alcohol consumption, less physical activity, less time spent on hobbies, and a poorer economic status. Additionally, differences emerged in COVID−19−related information and protective behaviors. Individuals with depressive symptoms were less likely to obtain news from national media, reported paying greater attention to COVID−19 news, and did not adopt significantly more protective measures than before the pandemic. The regression model, which included psychological (PSS score, UCLA Loneliness Scale score), sleep (difficulty in falling asleep, early awakening, sleep duration), and exercise (days of anaerobic exercise, aerobic exercise, and slow walking) factors, explained 53.9% of the variance in depressive symptoms among older adults, and the findings were statistically validated to be robust. Collectively, these findings suggest that multiple lifestyle and psychosocial changes during the pandemic were associated with depressive symptoms in older adults, highlighting potential targets for timely interventions.

Previous studies on sleep during the pandemic have yielded mixed results. While some studies have reported increased sleep duration (13, 21), others have observed an increase in insomnia, which is significantly associated with psychological distress, depression, anxiety, and financial stress (22, 23). Consistent with the latter, our study found a significantly higher incidence of difficulty in falling asleep and early awakening after the COVID−19 outbreak compared to the pre-pandemic period. Furthermore, older adults with depression exhibited higher rates of sleep disturbances and shorter sleep durations than their non-depressed counterparts. Regression analysis confirmed that sleep difficulties, including difficulty in falling asleep, early awakening, and shorter sleep duration, were strongly associated with higher depression scores. A review by Cipriani et al. (24) also noted significant links between sleep problems and mood disorders, particularly depression and anxiety. Taken together, these results indicate that multiple types of sleep disturbances exacerbated by the pandemic are important risk factors for depression in older adults. Addressing these sleep issues, perhaps through telemedicine or brief behavioral interventions for insomnia, might be a crucial component of mental health support for this age group.

Exercise helps alleviate depressive symptoms, and this holds true for older adults as well (25, 26). During the pandemic, people’s opportunities for exercise were restricted; consequently, the reduction in physical activity was associated with an increase in depressive symptoms among older adults. Our study confirmed that decreased levels of aerobic exercise, anaerobic exercise, and even slow walking were all related to the rise in depressive symptoms in older adults during the COVID-19 pandemic. A study from Japan found that engaging in physical activity during the COVID-19 lockdown helped prevent depressive symptoms (27). Collectively, these findings underscore that promoting safe forms of physical activity, irrespective of type, is a viable strategy to mitigate depression risk in older adults during public health crises. We also observed elevated stress and loneliness scores after the pandemic onset, both of which were strongly associated with depression levels. Increased stress may be attributed to multiple pandemic-related factors, including lifestyle disruptions (28), worsening economic conditions (29), fear of infection, social isolation, quarantine, stigma, and information overload (30). Older adults, who faced the highest mortality risk from COVID−19 (31), may have been particularly vulnerable to psychological distress. Loneliness, which tends to increase with age (32), was further amplified by the pandemic-imposed social restrictions (33). Creese et al. (14) identified loneliness and reduced physical activity as key risk factors for worsening mental health during this period. Thus, both stress and loneliness have emerged as significant modifiable risk factors for depression in older adults, suggesting that interventions aimed at mitigating loneliness may help reduce depressive symptoms (34) and lower all−cause mortality (35).

Exposure to COVID-19-related news, particularly via social media, has been linked to adverse mental health outcomes (36). However, our findings, along with the results of other studies (37, 38), suggest a more nuanced picture. While older adults with depressive symptoms reported paying greater attention to COVID-19 news, they were less likely to use trusted national media sources. This paradoxical finding—high engagement but low trust in authoritative sources—may contribute to confusion and distress, especially if they are not adopting effective protective behaviors. Indeed, our data show that the depression group did not adopt significantly more protective measures, suggesting a disconnect between information consumption and actionable health behaviors. This highlights the critical need for public health messaging to be both accessible through trusted channels (e.g., national radio) and empowering, to translate information into clear, actionable steps.

### 4.1 Limitations

Several limitations should be considered when interpreting our findings. First, while the CESD-8 is a well-validated screening tool for large-scale surveys, it is not a substitute for a clinical diagnostic interview for depression. Second, our study design has inherent limitations. The samples from Wave 5 and the COVID-19 Sub-Study were not identical, which limits the comparability of pre- and post-pandemic estimates. Furthermore, the absence of pre-pandemic (Wave 5) depression data for the COVID-19 sample precludes any causal inference regarding the effects of the pandemic on individual-level changes in mental health. Our cross-sectional analysis of the COVID-19 sample can only identify correlates, not determine whether the observed factors preceded or were consequences of depressive symptoms. Third, by excluding participants who reported a COVID-19 infection, we may have inadvertently introduced selection bias, limiting the generalizability of our findings to the entire population of older adults. Finally, the long-term mental health trajectory following the pandemic warrants further investigation in future follow-up studies.

## 5 Conclusion

This study demonstrated a significant increase in depression among older adults during the COVID−19 pandemic, which was associated with adverse lifestyle changes, reduced physical activity, sleep disturbances, increased loneliness and stress, and altered patterns of news consumption and protective behavior. Future interventions aimed at promoting mental health in this population should address these modifiable risk factors. These findings have direct clinical and public health implications. For practitioners, they underscore the need to routinely screen older patients for sleep quality, loneliness, and physical activity levels as early indicators of depression risk. For public health specialists, they highlight the importance of designing multi-component interventions that promote sleep health, facilitate safe social connections and physical activity (e.g., community walking groups), and disseminate trustworthy information through channels older adults trust, such as the national radio. Addressing these specific, modifiable risk factors offers a clear and actionable pathway to protect and improve the mental well-being of the aging population during future health crises and in routine care.

## Data Availability

Publicly available datasets were analyzed in this study. This data can be found here: https://tilda.tcd.ie/data/accessing-data/.

## Appendix A. the 8-item Center for Epidemiologic Studies Depression Scale (CESD-8).

For each item in the list below, please indicate how often you have felt or behaved this way during the last 7 days? Answer categories are: Rarely or none of the time (less than 1 day), Some or a little of the time (1–2 days), Occasionally or a moderate amount of time (3–4 days), All of the time (5–7 days).

- I felt depressed
- I felt that everything I did was an effort
- My sleep was restless
- I was happy
- I felt lonely
- I enjoyed life
- I felt sad
- I could not get “going”

### Appendix B. the UCLA Loneliness Scale

The next questions are about how you felt about different aspects of your life during the COVID-19 pandemic. For each one, please say how often you felt that way. Answer categories are: Often, Some of the time, Hardly ever or never.

- How often do you feel you lack companionship?
- How often do you feel left out?
- How often do you feel isolated from others?
- How often do you feel in tune with the people around you?
- How often do you feel lonely?

### Appendix C. the 4-item Perceived Stress Scale (PSS-4)

The next four questions are also about how you have felt during the COVID-19 pandemic. Answer categories are: Hardly ever, Almost never, Sometimes, Fairly often, Very often.

- How often have you felt that you were unable to control the important things in your life?
- How often have you felt confident about your ability to handle your personal problems?
- How often have you felt that things were going your way?
- How often have you felt difficulties were piling up so high that you could not overcome them?

## Appendix B. the UCLA Loneliness Scale

The next questions are about how you felt about different aspects of your life during the COVID-19 pandemic. For each one, please say how often you felt that way. Answer categories are: Often, Some of the time, Hardly ever or never.

- How often do you feel you lack companionship?
- How often do you feel left out?
- How often do you feel isolated from others?
- How often do you feel in tune with the people around you?
- How often do you feel lonely?

### Appendix C. the 4-item Perceived Stress Scale (PSS-4)

The next four questions are also about how you have felt during the COVID-19 pandemic. Answer categories are: Hardly ever, Almost never, Sometimes, Fairly often, Very often.

- How often have you felt that you were unable to control the important things in your life?
- How often have you felt confident about your ability to handle your personal problems?
- How often have you felt that things were going your way?
- How often have you felt difficulties were piling up so high that you could not overcome them?
